# A pull function attachment to manual wheelchairs: a case report on usability and shoulder pain in people with spinal cord injury

**DOI:** 10.1038/s41394-025-00704-2

**Published:** 2025-04-28

**Authors:** Mikkel Krogshede, Christoffer Holgaard, Joachim Alexander Valkær, Pascal Madeleine, Rasmus Kopp Hansen

**Affiliations:** 1 ExerciseTech, Department of Health Science and Technology, Aalborg University, Aalborg, Denmark; 2 Pull & GO I/S - Development of Health and Medical Equipment for Wheelchair Users, Aalborg, Denmark; 3 Respiratory and Critical Care Group, Department of Health Science and Technology, Aalborg University, Aalborg, Denmark

**Keywords:** Public health, Rehabilitation

## Abstract

**Introduction:**

Shoulder pain mostly due to high biomechanical load of the anterior-shoulder musculature is prevalent among manual wheelchair users with spinal cord injury or disorder (SCI/D). This case study showcases a novel pull-function attachment to the wheelchair that reverses the propulsion motion by pulling rather than pushing the wheelchair. Additionally, the study reports its preliminary usability and impact on shoulder pain.

**Case presentation:**

Four individuals with SCI/D (median age: 33; 1 female) used the novel pull-function attachment to the manual wheelchair for six weeks. Usability (System Usability Scale; ranging from 1 = ’strongly disagree’ to 5 = ’strongly agree’) and daily usage time were assessed. Shoulder pain intensity was explored before and after the six weeks using the Wheelchair User’s Shoulder Pain Index. Median usability scores were ≥3.0 for 5/6 items, indicating high usability of the assistive technology. Shoulder pain intensity was reduced in all participants at the post-test (median change: −6.3), with the change in shoulder pain exceeding the minimal detectable change for 2/3 participants.

**Discussion:**

The results of this case study suggest high usability of the pull-function attachment to the wheelchair. The decreases in shoulder pain intensity reported following six weeks of use were noticeable and may have clinical relevance. This novel assistive technology that enables forward propulsion by pulling rather than pushing the wheelchair may therefore have the potential to reduce shoulder pain in manual wheelchair users with SCI/D.

## Introduction

Approx. 10% of people with disability worldwide use a wheelchair for mobility [1] making wheelchairs one of the most commonly used assistive devices for mobility among people with disability, including those living with a spinal cord injury or disorder (SCI/D) [1, 2]. Although using a manual wheelchair allows for mobility, it often also impact musculoskeletal health [3]. Shoulder pain is commonly observed among manual wheelchair users with SCI/D, with prevalence rates reaching up to 76% [4], i.e., approximately three times the point prevalence in individuals without disability [5, 6]. Shoulder pain may negatively impact functional independence [7], physical activity levels [8], and ultimately quality of life [9]. Moreover, people with SCI/D experience shoulder pain as a limiting factor when performing activities of daily living (ADLs), such as wheelchair propulsion, transfers, reaching overhead, and lifting [10, 11]. Since manual wheelchair users are reliant on their upper extremities for mobility and ADLs [12, 13], these individuals, when in pain, cannot just simply avoid involvement of the shoulders to recover. Thus, preservation of shoulder function is of great importance [13]. Shoulder pain among manual wheelchair users with SCI/D is believed to be caused by the biomechanical load on the shoulder girdle [3, 14]. As using the shoulder joints becomes more painful, mobility can be impaired [7], thus reducing physical activity levels [8], resulting in a gradual decline in physical capacity and quality of life [9, 15].

Additionally, the sustained biomechanical load on the shoulder joint [14] associated with muscle strength imbalance may lead to rotator cuff impingement [16]. Wheelchair propulsion is a push-dominant movement [16], relying on the anterior muscles of the chest and shoulders [17]. Muscle strength imbalances have been reported in manual wheelchair users with SCI/D compared with people without disability. Here, it was demonstrated that the posterior muscles were relatively weaker than the anterior muscles when testing shoulder adduction and pulling strength [18]. Notably, strengthening the relatively weaker posterior muscles using scapular retraction exercises, as well as stretching the relatively stronger anterior muscles, have been shown to reduce shoulder pain among manual wheelchair users with SCI/D [19–21]. Therefore, exercises focusing on pull motions activating posterior muscles of the chest [18], might reduce shoulder pain over time.

Using mechanical add-on to the manual wheelchair, attempts have been made to reverse the propulsion pattern by converting the conventional wheelchair push-motion into a pulling motion [22], such as the Rowheel (Madison, Wisconsin, USA). Here, the drive wheel is swapped for the Rowheel drive wheels which enable the user to pull the drive rim on the wheelchair to create a forward motion [22]. This locomotion system, however, lacks flexibility as it only works by using the pull motion for forward propulsion when using the drive rim. To switch back to a push motion, and work the anterior muscles again, the Rowheel drive wheels will have to be swapped back to the standard drive wheels. Alternatively, a system that combines the pulling motion for forward propulsion, with the option of using the push motion, would be preferable to avoid overuse of the posterior muscles [23].

Our report showcases a novel pull-function attachment to the wheelchair that reverses the propulsion motion and reports its usability. We hypothesized that changing the direction of shoulder movement from push to pull will be usable and alleviate shoulder pain after six weeks of use.

## Case presentation

### Study design and participants

Four wheelchair-dependent participants with SCI/D were included in this case study (3 males, 1 female), all of which were provided with a pull-function attachment for their wheelchairs. A detailed description of participant characteristics is reported in Table 1. The inclusion of four participants was based on the number of manufactured pull attachment devices at the time of the study (*n* = 4). The participants were asked to maintain their usual living during the 6-week period and postpone any new exercise and/or physical activity programs until after the end of the study.

**Table 1:** Baseline demographic characteristics.

Participants	Sex	Age (yrs)	Height (m)	Weight (kg)	BMI (kg/m2)	SCI mechanism	NLI	AIS grade	Years in chair
P1	Male	47	1.69	53	18.6	SB	T2	A	36
P2	Male	18	1.65	70	25.7	SB	T9	B	18
P3	Male	50	1.98	114	29.1	SCI	T8	A	7
P4	Female	19	1.60	50	19.5	SCI	S2	C	3

*AIS* american spinal injury association impairment scale, *SB* spina bifida, *SCI* spinal cord injury, *NLI* Neurological level of injury.

Participants were recruited through multiple channels related to disability, parasport organizations, and SCI-patient organizations. The participants signed a Non-Disclosure Agreement regarding the product sensitivity and an acquiescence declaring confidentiality and well-informed consent. Inclusion criteria included using a manual wheelchair for mobility and having experienced shoulder pain within the last three months [24]. The three-month timeframe criterion aligns with the dynamic nature of shoulder pain, capturing participants in a period of ongoing discomfort and pain [25]. Participants were excluded if they suffered from any acute severe infection, inflammation, or illness/diagnosis. No participants have had any cortisol injections prior to the intervention. All participants provided written informed consent before participation in the study. Written informed consent was also obtained for publication of images of human research participants.

The study was conducted in accordance with the North Denmark Region Committee on Health Research Ethics (J.nr. 2-1-02-1167-24) and in agreement with the Helsinki Declaration.

### Intervention prescription

**Fig. 1:**
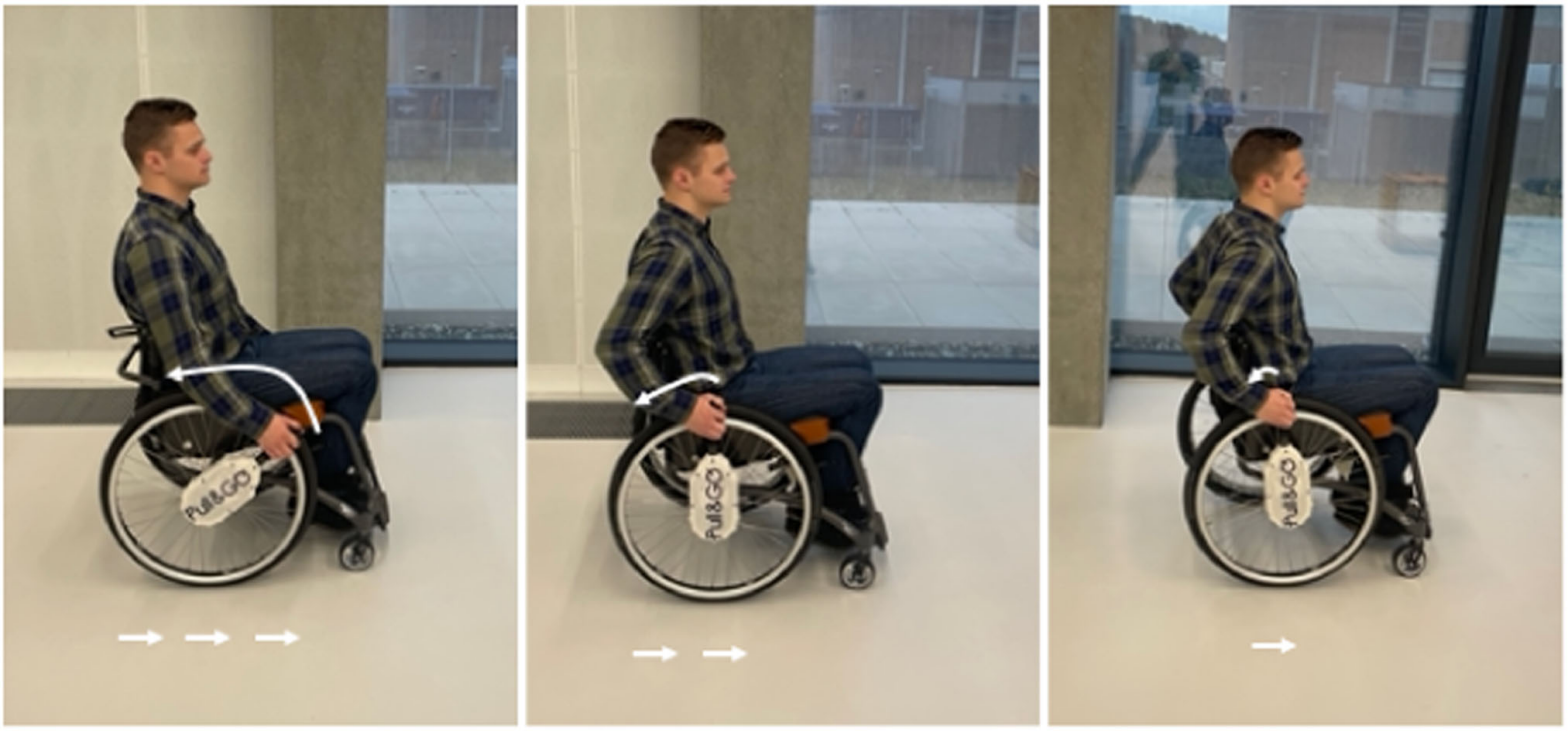
Pulling back on the handles creates forward movement of the wheelchair; while pushing forward on the handle, the wheelchair users will not encounter resistance but allow for continuation of pulling back in a “row-like motion” for forward propulsion.

The participants tested the attachment product from Pull & GO I/S (Aalborg East, North Jutland, Denmark). The product is called GO1 (GO series, version 1) utilizing a bevel gear system with a (1:1) ratio in propulsion speed allowing the user to bring the wheelchair into forward motion by pulling back on a handle using their arms. The GO1 is intended to relieve the load on the wrist and the shoulder joint compared with regular wheelchair propulsion (Fig. 1). The attachment is removable with magnet plates to easily attach and detach upon desire. Further, a saddle bag enables the user to carry the pull function around when not in use. The GO1 enables an easy shift between traditional wheelchair propulsion and pull propulsion at any time.

The pull function was installed on both sides of the participants’ wheelchairs. Participants were instructed about its usage and were encouraged to utilize the pull function during ADLs both outside and inside as much as possible.

### Data collection

At the beginning of the study, participants self-reported their age, body mass, height, disability etiology, and years of wheelchair use. Severity of the SCI (American Spinal Injury Association Impairment Scale) and neurological level of injury were determined by the International Standards for Neurological Classification of Spinal Cord Injury [26]. Shoulder pain intensity was assessed before (pre) and after the six weeks (post) with usability assessed only at the post-test. For assessment of shoulder pain intensity, participants were blinded to the scores reported at the pre-test.

#### Usability

The usability of the pull function attachment was assessed after the six weeks by System Usability Scale [27]. Participants were asked to score on a 5-point Likert scale the usability of the GO1 ranging from strongly disagree (1) to strongly agree (5), and to self-report their daily usage of the GO1 (Daily Time Period of the Pull Function [DTPPF]).

#### Wheelchair user’s shoulder pain index

Shoulder pain intensity was assessed using the Danish-translated version [28] of the Wheelchair User’s Shoulder Pain Index (WUSPI), a 15-item reliable and validated self-reported questionnaire [10, 29]. The questionnaire utilizes a 10-point visual analog scale (VAS) ranging from “no pain” to “worst pain ever experienced” during 15 different ADLs within the past week [10]. Briefly, the participants were asked to indicate the severity of shoulder pain while performing ADLs such as transfers, rolling up inclines, getting dressed, bathing, and sleeping. To adjust for any ADLs that was not performed, a performance-corrected score (PC-WUSPI) was calculated by dividing the raw total score by the number of executed activities and then multiplying by 15 [10].

Any adverse events were recorded. Data is presented using descriptive statistics (median [range]), with changes over time in shoulder pain reported as absolute changes.

## Results

One of the four participants (P1) dropped out halfway into the study due to a decubitus ulcer. Accordingly, *n* = 3 participants used the pull-function attachment for all six weeks.

### Usability of the pull-function attachment

**Fig. 2:**
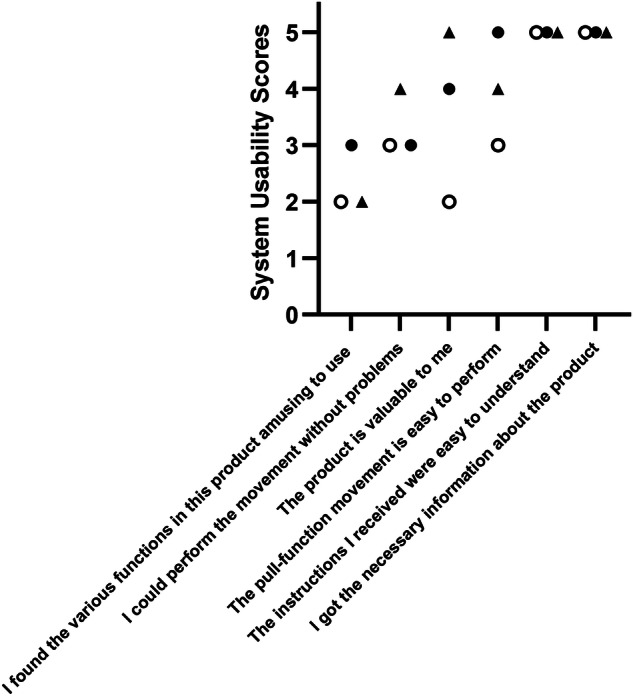
Individual participant system usability scores after the six weeks (*n* = 3). The six usability statements were scored on a 5-point Likert Scale, where 1 = strongly disagree and 5 = strongly agree. P2 = closed circles; P3 = open circles; P4 = triangles.

Individual system usability scale scores after the six weeks are presented in Fig. 2. Five out of six items on the usability scale had a median score of ≥3.0, suggesting high usability of the pull-function attachment, with two items reaching a score of 5 for all three participants (Fig. 2). In terms of adherence, the participants’ self-reported DTPPF was 0.32 (0.11–1.57) hours/day.

### Shoulder pain intensity

**Fig. 3:**
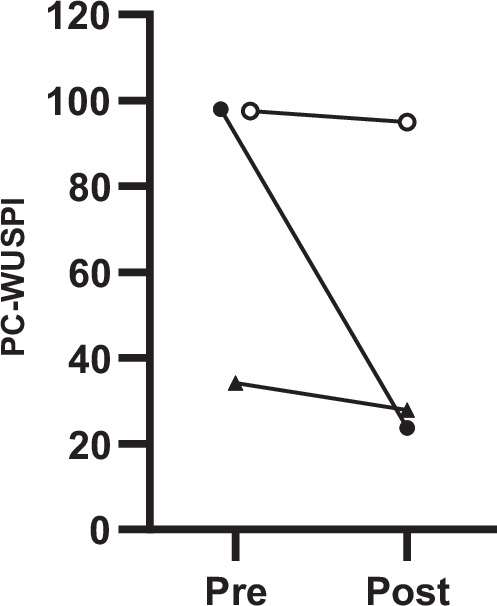
Individual participant changes in shoulder pain intensity from pre to post (*n* = 3). PC-WUSPI: performance corrected wheelchair user shoulder pain index. P2 = closed circles; P3 = open circles; P4 = triangles.

All three participants reported a reduction in shoulder pain intensity after the six weeks (Fig. 3). The absolute changes in pain intensity, as determined by PC-WUSPI, from pre- to post-test were −2.6 (P3); −6.3 (P4); and −74.2 (P2), respectively. No adverse events were reported.

## Discussion

This report showcased a novel assistive technology for the wheelchair enabling users to propel forward by pulling rather than pushing the wheelchair. Moreover, we assessed its preliminary usability as well as the impact on shoulder pain before and after six weeks of use. While caution to the interpretation of the findings is needed due to the nature of the study, our preliminary observations indicate that the pull-function attachment may be a useful add-on to the wheelchair with the potential to beneficially impact shoulder pain among manual wheelchair users with SCI/D.

People using wheelchairs face tremendous barriers to becoming physically active and/or sustaining physical activity. Some barriers do not change quickly, such as those belonging to the community-built environment [30]. Minimizing barriers related to the architecture and construction of the built environment may require interventions acting on a policy-level, which takes time [31]. Other prominent physical activity barriers among wheelchair users with SCI/D occur at an individual level and may be associated with shoulder pain. Identifying methods for reducing shoulder pain among manual wheelchair users with SCI/D is therefore an important area of research [32]. Physical activities that target posterior-shoulder muscles may be particularly beneficial in this population because of their potential to enhance muscle balance [19]. To our knowledge, a single study [22] has previously attempted to reverse the propulsion pattern using Rowheel drive wheels, thereby enhancing posterior-shoulder activation. Although innovative, this system lacks flexibility as the wheels are swapped and thus requires a permanent change in propulsion pattern.

In this report, we explored the preliminary usability of a detachable pull-function attachment that gives the user flexibility in terms of when to use the pull-function. Specifically, we utilized the System Usability Scale to assess the usability of the pull-function attachment, which allowed us to gain valuable insights into the users’ perceptions of the product’s functionality, value, ease of use, and the clarity of accompanying instructions. The System Usability Scale is a widely accepted and validated tool for evaluating the usability of various systems and products [33]. A median score of ≥3.0 has previously been used as a cutoff to indicate usability and acceptability of adaptive equipment in wheelchair users with SCI/D [34]. As five out of six items reached a median System Usability Scale score of at least 3.0, our preliminary data therefore support the usability of the pull-function attachment in agreement with our hypothesis. Furthermore, the three users were overall satisfied with the product. Particularly high usability scores were provided for the items “*The instructions I received were easy to understand*” and *“I got the necessary information about the product”* (all participants scoring 5). This suggests that the accompanying guidance for using the pull-function attachment was effective, contributing to users’ confidence and competence in utilizing the product. Clear and comprehensive information about any new product is critical for ensuring that users are well-informed about the features and benefits of the product. Indeed, adequate information fosters user understanding, which is pivotal for the successful adoption and sustained use of assistive technologies [33]. While the System Usability Scale scores suggest high usability, it should be noted that adherence to the pull-function attachment was only 0.32 h/day as quantified by the DTPPF. Such relatively low usage time needs to be considered when interpreting the applicability of the product. It is unclear whether the relatively low usage time was because the participants found the product uncomfortable to use or whether it simply reflects that the product was unaccustomed for the participants. In this regard, a longer intervention period allowing more time for participants to be accustomed could have been preferable. In light of the usability scores, it is unlikely that the participants were unsatisfied with the product. Some participants informed that it took time and practice to get accustomed to the product before mastering the pulling propulsion resulting in increased usage at the end of the study period. Taken together, while our preliminary data support the usability of the pull-function attachment, more research is needed to further explore the usability and satisfaction of this new assistive technology.

Despite relying on descriptive statistics and a small number of participants, there were some noteworthy observations related to changes in shoulder pain intensity over the six weeks. As hypothesized, all participants completing the study reported a reduction in shoulder pain. When looking at the magnitude of the changes in PC-WUSPI, it is noteworthy that two out of three participants reported a reduction in pain beyond the minimal detectable change of 5.10 points, as suggested by Curtis et al. [29]. Although the minimal clinically important difference for this instrument has not yet been established, a change beyond the minimal detectable change may reflect a true and likely meaningful reduction in shoulder pain for the two participants. Interestingly, the largest decrease in pain was associated with the highest usability score (P2). While these observations need to be followed up by a larger study, our preliminary data indicate a potential of the pull-function attachment for beneficially impacting shoulder pain among manual wheelchair users with SCI/D. Such results align with previous studies emphasizing the importance of proper wheelchair design and functionalities in mitigating shoulder-related issues [34–37]. Although we did not confirm the presence of enhanced activation of the posterior-shoulder musculature by surface electromyography, it is reasonable to assume that the pull-attachment resulted in higher motor variability [38] due to higher level of activation of the posterior muscle groups (including the lower trapezius, rhomboids, and latissimus dorsi), as well as shoulder external rotators. These muscle groups are important for reducing shoulder pain and balancing the stronger anterior-shoulder muscles [39]. Future studies are encouraged to include surface electromyography measurements to confirm this assumption.

### Limitations

There are some limitations associated with this case report. The few participants warrant some caution in the interpretation and generalization of the results. While a larger sample size would have been preferable, the recruitment was limited by the fact that only four pull-function attachments were available.

In conclusion, this case report showcased a novel assistive technology for manual wheelchair users with SCI/D that, in contrast to the conventional push-motion, enables forward propulsion of the wheelchair by pulling on an attachment placed on the wheels of the chair. The high usability scores support the preliminary usability of the product, although the limited usage time warrants further investigation. The individual changes in shoulder pain intensity highlight the pull-function attachment as a potential tool to reduce or prevent shoulder pain among manual wheelchair users with SCI/D. Further research with larger sample sizes is warranted to confirm these preliminary findings and explore additional outcomes.

## Data Availability

The data sets generated and/or analyzed during the current study are available from the corresponding author upon reasonable request.
